# General anaesthesia related mortality in a limited resource settings region: a retrospective study in two teaching hospitals of Butembo

**DOI:** 10.1186/s12871-021-01280-2

**Published:** 2021-02-23

**Authors:** Furaha Nzanzu Blaise Pascal, Agnes Malisawa, Andreas Barratt-Due, Felix Namboya, Gregor Pollach

**Affiliations:** 1 Department of Anaesthesia and Intensive Care, College of Medicine, University of Malawi, Blantyre, Malawi; 2 Faculty of Medicine, Université Catholique du Graben, Butembo, Democratic Republic of the Congo; 3 Matanda Hospital of Butembo, Butembo, Democratic Republic of the Congo; 4 Division of Emergencies and Critical Care, Rikshospitalet, Oslo University Hospital, Oslo, Norway

## Abstract

**Background:**

General anaesthesia (GA) in developing countries is still a high-risk practice, especially in Africa, accompanied with high morbidity and mortality. No study has yet been conducted in Butembo in the Democratic Republic of the Congo to determine the mortality related to GA practice. The main objective of this study was to assess mortality related to GA in Butembo.

**Methods:**

This was a retrospective descriptive and analytic study of patients who underwent surgery under GA in the 2 main teaching hospitals of Butembo from January 2011 to December 2015. Data were collected from patients files, anaesthesia registries and were analysed with SPSS 26.

**Results:**

From a total of 921 patients, 539 (58.5%) were male and 382 (41.5%) female patients. A total of 83 (9.0%) patients died representing an overall perioperative mortality rate of 90 per 1000. Out of the 83 deaths, 38 occurred within 24 h representing GA related mortality of 41 per 1000. There was a global drop in mortality from 2011 to 2015. The risk factors of death were: being a neonate or a senior adult, emergency operation, ASA physical status > 2 and a single deranged vital sign preoperatively, presenting any complication during GA, anaesthesia duration > 120 minutes as well as visceral surgeries/laparotomies. Ketamine was the most employed anaesthetic.

**Conclusion:**

GA related mortality is very high in Butembo. Improved GA services and outcomes can be obtained by training more anaesthesia providers, proper patients monitoring, improved infrastructure, better equipment and drugs procurement and considering regional anaesthesia whenever possible.

## Background

General anaesthesia (GA) in developing countries is still a high-risk practice [1–3]. The practice faces numerous challenges exclusively related to high number of pathologies, shortage of material and drugs, infrastructure and human resources [2, 4, 5]. In addition, a very dysfunctional health system is worsening the situation in Sub-Saharan Africa. However, the presence of adequate infrastructure, skilled anaesthesia providers and the use of effective sanitation are paramount to improve the anaesthesia outcome [6–8].

Several investigators have reported that anaesthesia-related morbidity and mortality rates have declined overtime, which have been attributed to a variety of safety improvements. This include advances in training, improved monitoring techniques, development and widespread adoption of practice evidence-based guidelines, and other systematic approaches to error reduction such as checklists and procedures protocols, airway management tools, sharing of safety knowledge and peer review, labelling of drugs, teamwork and simulation [7, 9–13].

A comparison of the reports from different countries in the period 1954–1989 with 1990–2006 reflects a decrease in anaesthesia-related mortality rates from 0.03–0.79 per 1000 anaesthetics to 0.01–0.57 per 1000 anaesthetics in developed countries. Studies from 2010 to date have reported similar rates with variations from country to country [2, 14–18].

However, in developing countries of Africa, mortality rates are still higher than in developed countries [6, 7, 11, 19–22]. The downward trend observed worldwide seems not to be as effective in Africa. For instance, in Zambia, avoidable mortality rate was still unchanged (32%) from 1989 to 2012 [23, 24]. In Malawi in 2000, Hansen et al. reported 51 complications and 14 deaths in 3022 anaesthetics during a period of 6 months. Eleven of the 14 deaths were identified as avoidable with an avoidable mortality rate (AMR) of 1:275. Considering factors of these deaths, anaesthesia-related mortality represented an AMR of 1:504 which was higher than in developed countries [20]. The intraoperative mortality rate in Malawi is still high and had not improved over time when comparing data from 2004 to 2006 with 2015–2016 [25]. In Ivory Coast during the same period, the mortality was 3.9 per 1000 patients who were anesthetized and was still very high [21]. In Togo, the 24 h mortality rate including all causes was 25.7 per 1000 anaesthetics, with anaesthesia AMR of 1:133 in 2002. It decreased to 8.9 per 1000 in 2006. The drop was associated with increased number of physician providers, initiation of anaesthesia preoperative clinics, creation of postoperative anaesthesia care unit (PACU) and practice of loco-regional anaesthesia [26, 27].

In the Democratic Republic of the Congo (DRC), Davies et al. reported a 48 h anaesthesia mortality rate of 8.67 per 1000 which was less than the rates observed in South Soudan (17.81) and in the Central African Republic (15.20) [28]. Studies conducted in obstetrics have reported high rates of maternal and neonatal mortality directly or indirectly associated with anaesthesia [29, 30].

GA remains widely used in hospitals in the DRC. According to Ahuka OL, up to 35.2% of surgeries are done under GA in the DRC and it is likely to be the same in Butembo, a City in the North Kivu Province in the eastern part of the DRC [31]. Morbidity and mortality related to GA directly or indirectly remain high in the DRC [28, 30, 31]. Although the documentation from general surgery are scarce, a few obstetrics studies largely prove this situation. Maternal mortality rate of up to 20 for 1000 associated directly or partially have been reported [29]. Furaha et al. conducted a 3 years study (2011 to 2013) about maternal mortality in four hospitals directed by the “Bureau Diocésain des oeuvres médicales” (BDOM) within 2 hospitals in Butembo and 2 others around Butembo. They found that 9 out of 14 maternal deaths that occurred in 56 women with complications after caesarean section were associated with GA [30].

Furthermore, GA is a veritable problem in Butembo for several reasons. Lack of human resources is the major one. In the whole city of Butembo there is no physician specialized in anaesthesiology, and in practice, anaesthesia is managed either by ordinary nurses or surgeons. Nevertheless, few hospitals in the city have anaesthetic nurses. The other reasons are infrastructure constraints as well as lack of drugs, monitoring equipment and specific rooms for postoperative care. These problems, together, lead to unsafe and poor quality of anaesthesia services in Butembo. The aim of this study was to describe the practice of GA and determine the outcomes and risk factors of death of patients undergoing surgery under GA in Butembo.

## Methods

This research was conducted in the two main teaching hospitals associated with the “Université Catholique du Graben” (UCG) of Butembo City: the Matanda hospital of Butembo and, the “Cliniques Unversitaires du Graben” (CUG), both in North Kivu Province in DRC.

Butembo is a City of the North Kivu Region lying West of Ruwenzori Mount and Virunga National Park in the eastern part of the DRC. Butembo is at an altitude of 1736 m and close to the Equator line at latitude of 0°08′29″ North and longitude of 29°17′28″ East. Butembo has approximately 900,000 inhabitants.

This was a retrospective descriptive and analytic study of 5 years from January 2011 to December 2015. The population included all patients who underwent an operation under GA during the study period in the 2 hospitals.

The sample size was an exhaustive probability sampling using Slovin’s formula with confidence interval of 99%. All patients whose files were available, surgery or gynaecologic procedure performed under GA with American Society of Anesthesiologists Physical Status (ASA PS) equal or lower than 4 were included. Obstetric cases and patients of ASA PS 5 and above were exluded. Enquiries from the hospitals revealed that 1015 files of patients who received GA during the period of interest were available, of which 198 (19.5%) were from UCG and 817 (80.5%) from Matanda Hospital. A sample size of 921 was calculated and used. The sample was proportional to the cases found in each hospital and patients were systematically included in each sample proportion.

Data were collected using a data collection form designed for the study. Information was obtained from the anaesthetic registers, the anaesthetic personal file, and patient’s files. When a patient was operated on several times during the same hospital stay, only the last operation was included in the study whereas the other operations were considered as part of the history. Preoperative, intraoperative and postoperative variables were recorded.

Preoperative variables included patient-related variables such as age, gender, ASA PS, and surgery and anaesthetic histories, urgency of the procedure, pre-operative assessment, vital signs, and the date of admission. Intraoperative anaesthetic variables included the anaesthetic provider qualification and experience; type of anaesthetic agents used for induction; definitive airway management, type of agent used for maintenance, and surgical variables such as type of surgery, time and duration of the procedure. Postoperative variables included postoperative recovery location, complications after surgery, postoperative outcome, time and place of death, and date of discharge. Any anaesthetized patient who died during the surgery or within 24 h after the procedure, was defined as an “anaesthesia-related” death. No other causality was further sough to judge if the death was partially or fully associated to death. All patients who died after 24 h represented the “After 24h mortality”. The overall perioperative mortality was defined as all patients who died after a surgery under GA regardless the time.

Data were entered and analysed with SPSS Version 26. Descriptive statistics were used to describe variables. Logistic regression was used to determine the degree of association between the dependent variable (mortality) and independent variables (others variables). Associations were established using the Chi-square test of Pearson with a *p*-value below 0.05 considered as statistically significant. Odds ratio (OR) and/or Relative risk (RR) were calculated when necessary with their confidence intervals at 95% (95% CI).

This study was a low-risk research. The protocol was submitted and obtained approval from the “the Comité Ethique du Nord Kivu” (CENK) in Butembo in the DRC under number CENK N°007/2018 and from the College of Medicine Research Ethics Committee (COMREC) in Malawi under number P.07/18/2431. Participants’ consent was waived by both Ethics committees. The data were anonymous on the data collecting form and in the electronic Data Base. All research was performed in accordance with relevant guidelines/regulations in these hospitals.

## Results

**Fig. 1:**
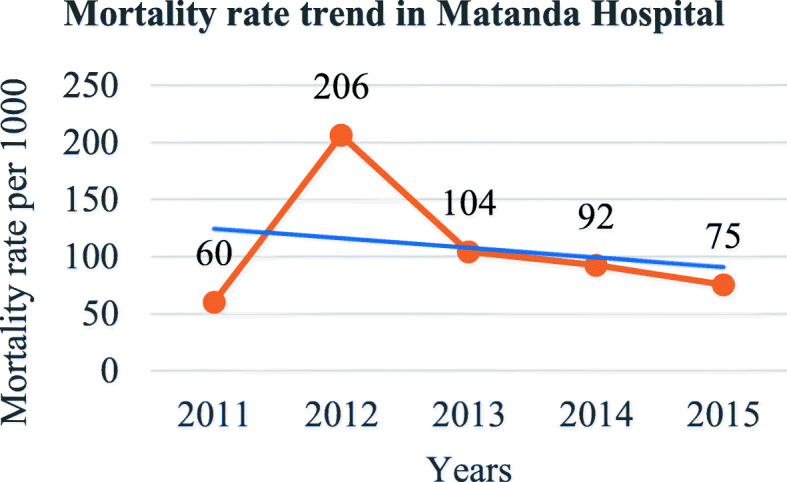
Overall mortality trend at Matanda Hospital

**Fig. 2:**
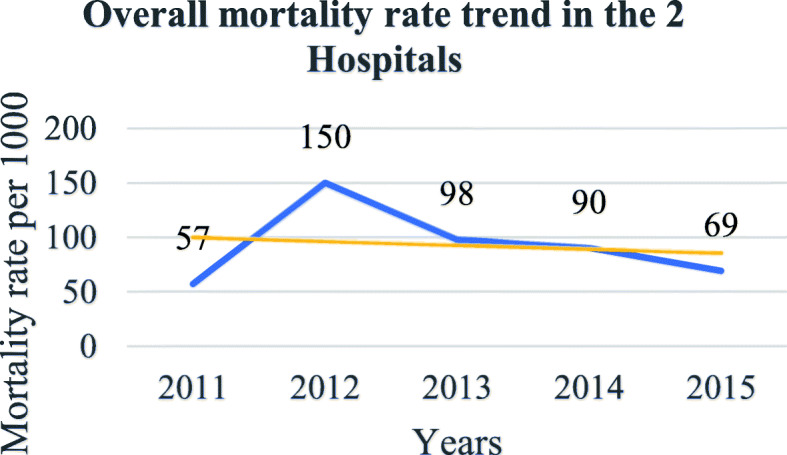
Overall mortality trend under GA in both hospitals

**Fig. 3:**
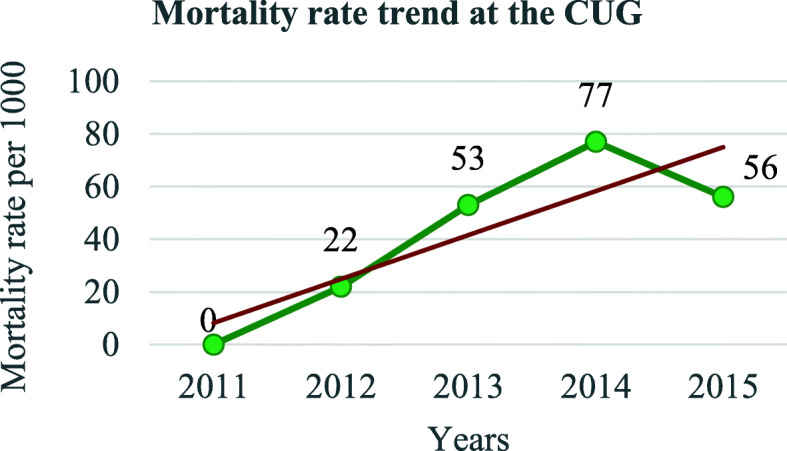
Overall mortality trend at CUG

In total, 921 patients were included the present study, 741(80.5%) from the Matanda hospital represented and 180(19.5%) from the CUG, altogether 539 (58.5%) males and 382 (41.5%) females. The sex ratio was 1.4 for males. A total of 83(9.0%) patients died which represented an overall perioperative mortality rate after a surgery under GA of 90 per 1000. From the 83 deaths, 38 deaths (45.8%) occurred within 24 h representing a GA related mortality (directly or indirectly) of 4.1% (38/921) or 41 per 1000. Four patients (4.8%) died at induction and 5 (6.0%) died during maintenance. Fifty-two patients (62.7%) died in the ICU against 1 in PACU (Table 1). There was a significant difference in places of death with more patients dying at the ICU (Hypothesis Test, *p* < 0.0001). There was a significant global drop trend in mortality from 2011 to 2015 (calculated χ2 = 9.80 (df = 4), *p* = 0.04) (Fig. 1). The trendline was significantly negative at the Matanda Hospital (χ2 = 16.75; df = 4; *p* = 0.002) (Fig. 2). The positive trendline observed at the CUG over years was not significant (χ2 = 1.64; df = 4; *p* = 0.80) (Fig. 3).

**Table 1:** Perioperative mortality under general anaesthesia

Issue	Patients (***N*** = 921)	%
Recovery	838	91.0
Death (Overall mortality)	83	9.0
**Mortality according to time**	***N*** **= 83**	
24H mortality (GA related mortality)	38	45.8
After 24H mortality	45	54.2
**Moment of Death**	***N*** **= 83**	
At induction	4	4.8
During maintenance	5	6.0
Postoperatively	74	89.2
**Place of death in the hospital**	***N*** **= 83**	
Intensive care	52	62.7
Patient Room/Ward	16	19.3
Operating Room	14	16.9
Postoperative care unit	1	1.2

Male patients died more frequently compared to female patients (*p* = 0.04). The median of age was 20 years with a minimum of 1 day and maximum of 90 years. There was a significant association between age and mortality (χ^2^ 43.14; *p* < 0.0001). The extreme ages were associated with increased risk of mortality. Neonates had 8.09 risk of dying compared to young adults (16-30 years old) (*p* < 0.0001). The risk of dying was increased by 3.08 for senior adults (≥51years old) compared to young adults (*p* = 0.002) (Table 2).

**Table 2:** Mortality according to gender and age of patients

Variables	Patients	%	Deaths (%)	X^**2**^	***p***-value	OR (95% CI)
**Gender**						
Male	539	58.5	57(10.6)	3.87	0.04	1.62(0.99–2.62)
Female	382	41.5	26(6.8)			1
**Total**	**921**	**100.0**	**83(9.0)**	**3.87**	**0.04**	
**Age group**						
0–30 days	29	3.1	10(34.5)	22.08	0.0001	8.03(3.02–21.39)
31 days-2 years	163	17.7	15(9.2)	1.54	0.29	1.54(0.68–3.47)
3–15 years	209	22.7	7(3.3)	1.70	0.19	0.52(0.20–1.39)
16–30 years	179	19.5	11(6.1)			1
31–50 years	204	22.1	17(8.3)	0.67	0.41	1.38(0.63–3.04)
≥ 51 years	137	14.9	23(16.8)	9.16	0.002	3.08(1.44–6.56)
**Total**	**921**	**100.0**	**83(9.0)**	**43.14**	**0.0001**	

The preoperative assessment was not done for 81 patients (8.8%) but this didn’t represent a factor of risk of mortality (*p* = 0.48). The emergency of the operation increased the risk by 4.48 (p < 0.0001) compared to non-emergent procedures. Previous surgery or GA were not associated with greater risk of death (*p* = 0.37 and *p* = 0.12 respectively). The risk of death increased significantly as ASA PS increased from 2 to 4. Patients with ASA PS 3 and ASA PS 4 had the highest risk of death. The presence of only one single deranged vital sign preoperatively represented 3.09 risk of death compared to patients with normal vital signs before operation (*p* < 0.0001) (Table 3). Premedication was given to 835(90.7%) patients and was primarily (85.1%) atropine based (atropine alone 75.5%, atropine+diazepam 9.1%, Atropine+other drugs 0.5%). Diazepam and midazolam alone were given as premedication to 3.6% and 1,8% patients respectively. Two patients received promethazine or dexamethasone as premedication drug whereas 9.3% of the patients did not receive any premedication.

**Table 3:** Mortality according to preoperative parameters and mortality

Parameters	Patients ***N*** = 921	%	Deaths (%)	X^**2**^	***p***-value	OR (95% CI)
**Pre-anaesthetic Assessment**						
Yes	840	91.2	74(8.8)			1
No	81	8.8	9(11.1)	0.5	0.48	1.29(0.62–2.69)
**History of surgery**						
Yes	324	35.2	33(10.1)	0.79	0.37	1.23(0.77–1.96)
No	597	64.8	50 (8.4)			1
**History of GA**						
Yes	265	28.8	30(11.2)	2.3	0.12	1.44(0.90–2.31)
No	656	71.2	53(8.1)			1
**Urgency of procedure**						
Emergency	198	21.5	42(21.2)	45.8	0.0001	4.48(2.81–7.12)
Non-Emergency	723	78.5	41(5.7)			1
**ASA Physical Status**						
ASA 1	378	41.0	5(1.3)			1
ASA 2	409	44.4	30(34.2)	16.7	0.0001	5.90(2.26–15.38)
ASA 3	111	12.1	38(34.2)	115.8	0.0001	38.83(14.78–101.98)
ASA 4	23	2.5	10(43.5)	107.0	0.0001	57.38(17.15–191.97)
**Vital signs**						
Normal	697	75.7	40(5.7)			1
Deranged	224	24.3	43(19.2)	37.4	0.0001	3.90(2.46–6.18)

Trained anaesthetic nurses conducted 873(94.8%) anaesthesia procedures. They were involved in 81(97.5%) deaths of the 83 deaths. The qualification of the anaesthesia provider was not associated with risk of dying in this study (*p* = 0.59). Anaesthesia providers with experience of more than 5 years conducted 468(50.8%) anaesthesia procedures and were involved in 41(49.3%) of the 83 deaths. Experience was not associated with increased risk of death (*p* = 0.37) (Table 4).

**Table 4:** Mortality according to qualification and experience of the anaesthesia provider

Parameters	Patients ***N*** = 921	%	Deaths (%)	X^**2**^	***p***-value	OR (95% CI)
**Anaesthesia Provider**						
Anaesthetic Nurse	873	94.8	81(9.3)			1
Any nurse	33	3.6	1(3.0)	1.50	0.21	0.30(0.04–2.26)
Trainee Anaesthetic nurse	11	1.2	1(9.1)	0.00	0.98	0.97(0.12–7.73)
Any Doctor	4	0.4	0			
**Provider work experience**						
Less than 1 year	45	4.9	5(11.1)	0.27	0.59	1.31(0.48–3.48)
1–2 years	189	20.5	22(11.6)	1.28	0.25	1.37(0.79–2.37)
3–4 years	219	23.8	15(6.8)	0.72	0.39	0.77(0.41–1.41)
Equal or more than 5 years	468	50.8	41(8.8)			1

Gas induction with halothane was given to 142(15.4%) patients of whom 21 died. Compared to patients who had a parenteral induction, gas induction increased the risk of death by 2.01 (*p* = 0.009). Ketamine was the agent of induction for 526 patients of 779(67.5%) parenteral inductions. Intubation was performed for 440(47.8%) patients and 52(62.7%) of 83 deaths occurred in intubated patients. Intubation increased the risk of death by 1.94 compared to non-intubated patients (*p* = 0.004) (Table 5).

**Table 5:** Factors of mortality at induction

Parameters	Patients (***N*** = 921)	%	Deaths (%)	X^**2**^	***p***-value	OR (95% CI)
**Route of GA induction**						
Parenteral (IV/IM)	779	84.6	62(7.9)			1
Inhalation	142	15.4	21(14.8)	6.83	0.009	2.01(1.18–3.41)
**IV/IM Anaesthetics**	***N*** **= 779**					
Ketamine	526	67.5	44(8.4)			
Thiopental	240	30.8	11(6.7)			
Propofol	13	1.7	2(15.4)			
**Airway Management**	***N*** **= 921**					
No intubation	481	52.2	31(6.4)			1
Oro-tracheal Intubation	440	47.8	52(11.8)	8.09	0.004	1.94(1.22–3.09)
**Complications at induction**	***N*** **= 789**					
Yes	24	3.0	8(33.3)	14.22	0.002	4.74(1.96–14.45)
No	765	97.0	73(9.5)			1
**Type of complications**						
None	765	97.0	73(9.5)			1
Hypotension	12	1.5	2(16.7)	0.68	0.41	1.89(0.41–8.81)
cardio-respiratory arrest	4	0.5	4(100)	36.13	0.0001	10.47(8.42–13.03)^a^
Vomiting	3	0.4	1(33.3)	1.94	0.16	4.73(0.42–17.56)
Difficult intubation	3	0.4	0(0)			
Cardiac arrest	1	0.1	1(100)	9.36	0.002	10.47(8.42–13.03)^a^
Hypertension	1	0.1	0(0)			

^a^Relative risk

Ketamine was the first drug used for maintenance of anaesthesia in 381 patients (41.4%) followed by halothane used for 227 patients (24.6%). Isoflurane, thiopental and propofol were used in 195(21.2%), 110(11.9%) and 8(0.9%) cases respectively. Gas maintenance of anaesthesia, performed in 422(45.8%) out of 921 patients, accounted for 47(11.1%) deaths in this group. Gas maintenance was associated with increased risk of mortality (OR = 1.61, *p* = 0.04) compared to iv maintenance. Complications during maintenance of anaesthesia were recorded in 14.4% of the patients (133/921), involving 33 patients (24.8%) and represented a highly increased risk of death (OR = 21.3, *p* < 0.0001). Hypotension and vomiting were the most frequent complications, both observed in 9 patients (6.8%), whereas only hypotension during maintenance was associated with increased mortality (OR = 24.5, *p* < 0.0001). Cardiac arrest and cardio-respiratory arrest were both fatal complications during maintenance of anaesthesia (Table 6).

**Table 6:** GA maintenance strategies, complications and mortality

Parameter	Patients	%	Deaths (%)	X^**2**^	***p***-value	OR (95% CI)
**Type of maintenance**						
Gas Maintenance	422	45.8	47(11.1)	4.29	0.04	1.61(1.02–2.54)
IV Maintenance	499	54.2	36(7.2)			1
**Maintenance complication**						
Yes	33	24.8	2(2.0)	24.2	0.0001	21.3(4.36–103.91)
No	100	75.2	10(30.3)			
**Type of complication**						
None	100	75.2	2(2.0)			1
Hypotension	9	6.8	3(33.3)	18.5	0.0001	24.50(3.41–175.67)
Vomiting	9	6.8	1(11.1)	2.6	0.10	6.12(0.49–75.09)
Hypertension	6	4.5	1(16.5)	4.4	0.03	9.80(0.75–127.17)
Respiratory arrest	3	2.2	0(0)			
Cardio-respiratory arrest	3	2.2	3(100.0)	60.6	0.0001	50.00(12.68–197.16)^*^
Cardiac arrest	2	1.5	2(100.0)	49.9	0.0001	50.00(12.68–197.16)^*^
Accidental extubation	1	0.8	0(0)			

The mean duration of anaesthesia was 81.1 min with a standard deviation of 50.8 min (min. 10, max. 360 min). Duration of anaesthesia was associated with increased risk of death (χ^2^ = 39.1; df = 3; *p* < 0.0001) with a higher risk of death the longer the procedure lasted. The type of surgery performed was significantly associated with risk of death ((χ^2^ = 96.5; df = 10; *p* < 0.0001). Visceral surgeries/laparotomies increased the risk of patient death compared to orthopaedic surgeries (OR = 7.3, p = < 0.0001) (Table 7).

**Table 7:** Mortality according to duration of anaesthesia and type of surgery

Parameters	Patients ***N*** = 921	%	Deaths (%)	X^**2**^	***p***-value	OR (95% CI)
**Duration of Anaesthesia** (Minutes)						
≤ 60	441	47.9	24(5.4)			1
61–120	341	37.0	28(8.2)	2.37	0.12	1.6(0.9–2.7)
121–150	91	9.9	18(19.8)	21.32	0.000	4.3(2.2–8.3)
> 150	48	5.2	13(27.1)	28.98	0.000	6.6(3.0–13.8)
**Type of Surgery**						
Visceral surgery/laparotomy	304	33,0	64(21.0)	22.71	0.000	7.3(2.9–18.6)
Orthopaedic	142	15,4	5(3.5)			1
ENT	126	13,7	1(0.8)	2.66	0.13	0.2(0.0–1.9)
Hernia	124	13,5	1(0.8)	2.21	0.13	0.2(0.0–1.9)
Others	78	8,5	4(5.1)	0.33	0.56	1.5(0.3–5.6)
Plastics	43	4,7	1(2.3)	0.15	0.69	0.6(0.1–5.4)
Gynaecology	34	3,7	0(0)			
Urology	26	2,8	0(0)			
Ano-rectal malformations	26	2,8	6(23.1)	13.73	0.000	8.2(2.3–29.5)
EUA	9	1,0	0(0)			
Neurology	9	1,0	1(11.1)	1.27	0.25	3.4(0.4–32.9)

**Fig. 4:**
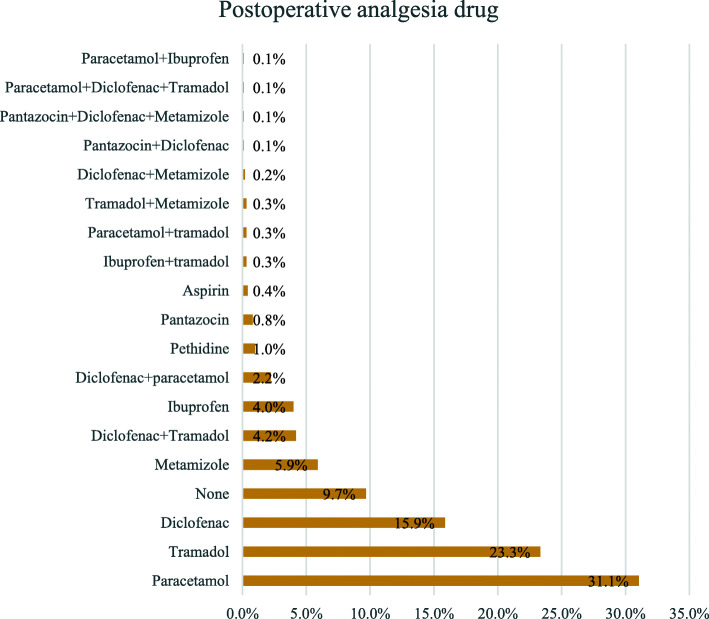
Bar graph of postoperative analgesia drugs

Postoperative vital signs were deranged for 324(35.5%) patients. Deranged postoperative vital signs increased the risk of death by 4.98 (*p* = 0.0001). A total of 500(64.5%) patients were admitted directly in their wards after operation. Nine patients (1%) were left in theatre postoperatively. There was an association between the recovery location and death (χ^2^ = 102.68; df = 3; *p* = 0.0001). Patients having their recovery in theatre had 76 times as much chance of dying compared to patients who were admitted in PACU postoperatively. Postoperative complications for the 912 patients who reached the postoperative period were presented among 159 patients representing a postoperative morbidity rate of 17.4% and were highly associated with increased mortality (χ^2^ = 310.17; df = 1; OR = 93.03; *p* < 0.0001) (Table 8). Paracetamol alone was the most employed drug for postoperative pain control followed by the combination of paracetamol and diclofenac injection. Metamizole was used for 6.2% of patients (Fig. 4).

**Table 8:** Postoperative complications and death

Postoperative parameters	Patients ***N*** = 912	%	Deaths (%)	X^**2**^	***p***-value	OR (95% CI)
**Vital signs**						
Normal	597	64.8	25(4.2)			1
Deranged	324	35.2	58(17.9)	48.16	0.000	4.98(3.05–8.15)
**Recovery location**						
Ward	500	54.3	17(3.4)	2.15	0.14	0.33(0.07–1.55)
Intensive care	391	42.4	56(14.3)	0.37	0.53	1.58(0.36–7.00)
PACU	21	2.3	2(9.5)			1
Operating room	9	1.0	8(88.9)	17.85	0.0001	76.00(6.00–962.32)
**Postoperative complication**						
yes	159	17.4	68(42.8)	310.17	0.0001	93.03(39.27–220.42)
No	753	82.6	6(0.8)	310.17	0.0001	1
**Type of complications**						
None	753	82.6	6(0.8)	697.64		1
Hyperthermia	49	5.4	7(14.3)	17	0.0001	20.75(6.67–64.48)
Coma	36	3.9	36(100)	670.89	0.0001	125.5(59.56–278.46)^a^
Nausea and vomiting	34	3.7	4(11.8)	31.19	0.0001	16.6(4.44–61.93)
Sepsis	14	1.5	14(100)	532.62	0.0001	125.5(59.56–278.46)^a^
Cardiorespiratory arrest	3	0.3	3(100)	249.99	0.0001	125.5(59.56–278.46)
Anaemia	2	0.2	1(50)	52.57	0.0001	24.50(6.95–2231.07)
Aspiration pneumonia	2	0.2	2(100)	187.24	0.0001	125.5(59.56–278.46)^a^
Respiratory arrest	1	0.1	1(100)	125.50	0.0001	125.5(59.56–278.46)^a^
Others^b^	18	1.3	0(0)			

^a^Relative risk

^b^Others (Vertigo 7, sore throat 3, Hypertension 2, Logorrhea 2, Neck pain 1, Hiccups 1, Epistaxis 1, Hypothermia 1)

## Discussion

This study described GA related mortality among patients undergoing surgery in Butembo. For our knowledge, this is the first study to evaluate the practice of GA in its all aspects in the region. As a baseline study, it elucidates the situation and the practice of GA in the region, the profile of patients, the anaesthesia providers’ qualifications and experience and the common drugs used. Additionally, this study documents the frequent complications and the outcome of patients operated under GA. Importantly, the study reveals insufficient areas of provided anaesthesia services, key elements that need to be addressed when planning for an improved future anaesthesia practice in the region.

From this study, 83 patients (9.0%) died after an operation under GA, which represented an overall perioperative mortality rate after GA of 90 per 1000. According to the definition in this study, GA was implicated directly or indirectly in 38 of the 83 deaths (45.8%) who died within 24 h, representing a GA related mortality rate of 4.1% or 41 per 1000 procedures. Both the overall perioperative and the GA related mortality rates were very high. However, there was an overall decrease mortality over time reflecting an improvement that may be related to gained experience overtime by providers and employment of qualified anaesthetic nurses since the infrastructure was almost the same. Other reasons may additionally explain this improvement, but would require different and more data and is either not the scope of this study. The findings of this study are consistent with trends in decreased mortality observed in Africa and worldwide [2, 32–35]. However, efforts should be made to further lowering the mortality.

Few studies have reported specific mortality after a GA in developing countries but a lot of studies exist on perioperative mortality. Similar to the results in this study, Rickard et al. in Rwanda reported an overall perioperative mortality of 6.5% and the 24 h mortality of 35% [22]. Ariyamata et al., in their study in high, middle and low income countries, reported a perioperative mortality within 24 h between 8 and 20% higher than in Togo, Kenya, Zambia and in the African Surgical Outcomes study (ASOS) but lower than observed in our study [24, 26, 27, 33, 34, 36]. In the European Surgical Outcomes Study (EuSOS), Pearse et al. noted a 7 days’ mortality of 4% with wide variability between countries (1.2% in Ireland and 21.5% in Latvia) [35]. The high rate observed in this study might be due the fact that the hospitals in this study are poorly equipped and lack human resources. Non-trained providers were administrating GA. Drugs supply is also questionable. None of the hospitals had a fully working anaesthesia machine during the period of study. A recent study done by Blaise Pascal et al. on practice of standard monitoring in the same region support these hypotheses [37]. Lack of proper infrastructure, drugs, equipment and trained providers are facts limiting safe anaesthesia [38, 39]. Efforts should then be made by these hospitals to procure all the necessary drugs and equipment, to improve the infrastructure and to employ trained providers or participate in their training for safe GA.

Almost 10.6% of deaths (9 patients) occurred intraoperatively. The high intraoperative rate in this study corroborates the results observed by Prin et al. in Malawi [25]. This raised a question on the level of knowledge and skills of anaesthesia providers on management of intraoperative emergencies such as cardiac arrest which was fatal in this study and obviously a need to empower them with adequate knowledge and skills in cardiorespiratory resuscitation and intraoperative emergencies management.

This study also found that a big number of patients that died in the ICU postoperatively. In the ASOS, Biccard et al. observed a high mortality rate in patients admitted in ICU postoperatively compared to those who were not admitted [34]. High mortality in ICU can be explained by the fact that both hospitals didn’t have proper ICU facilities. In the settings of this study, all so-called ICUs were under equipped. They didn’t have ventilators, monitoring, 100% oxygen, syringe-pumps as well as other important devices for ICU. Strictly speaking they were more like high dependency units. Patients who require ICU admission are usually very sick, implying that a low setting ICU represents an additional risk which may impact their outcome negatively [6, 7, 40].

This study demonstrated that age, especially extreme ages like neonates and senior adults, were significantly associated with increased death. This coincides with several reports recognizing extreme age as a factor of perioperative mortality [2, 4, 6, 22, 28, 34, 35, 41, 42]. The high number of deaths in the neonatal group, with extension to the paediatric, expresses the poor quality of paediatric anaesthesia in the region and a need to implement changes to reduce mortality in this group. This can be achieved by strengthening the available workforce in paediatric anaesthesia by refresher courses and seminars in paediatric anaesthesia, training of more anaesthesia providers and improvement of the infrastructure.

Emergency procedures increased the risk of death and corroborates the results from Europe, Australia and low- income and middle-income countries [32, 35, 43]. Emergency patients are most of the time very sick or with a life-threatening condition that increased their mortality risk [2, 12, 34, 44]. Furthermore, emergency patients are often not well optimized before surgery especially in low-income countries where drugs are not available all the time. Poor preoperative optimization has been found associated with high mortality [35, 45, 46]. In this study, the poor preoperative optimization state correlated to the presence of one single deranged vital sign preoperatively and was highly associated with increased mortality risk. Similarly, Hollis et al. identified deranged vital signs as a factor of Critical Postoperative Complications [47]. Deranged vital signs at admission, used in systematic scores like in the Early Warning Score (EWS), have been associated with increased death in the ICU, the emergency departments as well as in surgery [48–52]. In low-income countries, access to sophisticated preoperative laboratory tests and imaging is challenging, thus underscoring the vital importance of using the EWS systematically. This is a simple approach, easy and cheap and may help the clinician to detect patient deterioration and guide the anaesthesia plan. However, since this study didn’t evaluate the EWS specifically, further prospective studies are needed to confirm EWS would be an important of EWS preoperative assessment tool in anaesthesia.

Increased ASA PS correlates with high mortality risk [2, 22, 24, 25, 34, 35]. The present study corroborates this, showing that ASA PS 3 and 4 patients were at higher risk of death. Special attention and optimization of these patients should be taken before any surgery to improve their outcome.

Neither the qualification nor the experience of the anaesthesia providers was associated with death. In comparison to this study, the Cochrane review done by Lewis et al. concluded that it was not possible to draw a clear decision whether a physician versus non -physician anaesthesia providers, had any impact on safe anaesthesia and outcome [53].

This study revealed that gas induction and intubation multiplied the risk of dying by a factor of 2 and 1.9 respectively. This is consistent with a report documenting from 21 countries with resource-limited settings, that GA without intubation was associated with lower mortality compared to GA with intubation [54]. The anaesthesia settings of the hospitals in this study were lower. All hospitals didn’t have proper anaesthesia machines with mechanical ventilation, proper system of oxygen procurement, drugs and good monitoring. In such situations, intubating a patient represents a high risk of hypoxia, hypoventilation, hypercapnia and all other cascades which ineluctably may lead to death [6–8, 12]. Furthermore, in this study, gas maintenance of anaesthesia carried higher risk of death compared to iv maintenance. This situation illustrates once more a problem with management of intubated patients. In general, Ketamine was the most used drug for iv induction and maintenance. This might have explained the large use of atropine as a premedication drug. In Africa, Ketamine is the most available drugs, and because of its properties it is easy to use even by non-trained providers. Ketamine anaesthesia allows maintenance of spontaneous respiration, and BP with slight increase in BP and provides analgesia [27, 33, 54–56]. Thus, in limited setting conditions, for procedures where the risk of aspiration is not very high, it could be much better to keep the patient spontaneously breathing without intubation. This is often successfully achieved with ketamine. Contrary, intubation of a patient requires in general more resources, equipment, monitoring and good skills for a safe technique. Intubation may become hazardous and increase the risk of unwanted events and death if these prerequires are not met. Further studies are needed to confirm the safety of Ketamine use in lower setting conditions. Additionally, in order to better take care of intubated patient, which is the gold standard for GA, and reduce the risk under GA with intubation, hospitals and all stakeholders should include training of anaesthesia providers in their plan as well as procurement of equipment, drugs and oxygen for safe anaesthesia and surgery. Employing regional anaesthesia could also help to avoid unsafe general anaesthesia [9, 15, 16, 18].

Occurrence of any complication at induction or during maintenance of anaesthesia was significantly associated with increased risk of death. These results corroborate the findings of the International Surgical Outcomes Study [57].

Patients who had a laparotomy/visceral surgery and patients with anal malformations presented high risk of death compared to orthopaedics patients. Laparotomy/visceral surgery has been identified as a factor of death in several studies [4, 27, 32, 33, 57, 58]. Laparotomy/visceral surgery patients are often very sick patients. The patients in the present study were operated under non-optimal GA, primary due to lack of resources. They were often not intubated implying increased risk of aspiration and aspiration pneumonia. Whenever they were intubated proper mechanical ventilation and proper monitoring were not used. All those conditions are well known associated with high mortality [12, 25, 34, 59].

Long duration of procedure (procedure more than 2 h) was associated with high mortality. Our results are similar to those obtained by Phan et al. who found significantly higher rates of any complication with prolonged anaesthesia [60]. As observed in this study, Cheng et al. in a systematic review and meta-analysis observed that the likelihood of complications increased significantly with prolonged operative duration, approximately doubling with operative time thresholds exceeding 2 or more hours [61].

A large number of patients (64.5%) were admitted directly in their wards from the operating room. Only 2.3% of patients were admitted in a PACU. This practice is unsafe since postoperative period is crucial for the safety of the patient and life-threatening complications happen during this period. Patients should be admitted in specific postoperative recovery given that PACU has help to improve outcome after surgery [38, 62]. Furthermore, patients left postoperatively in the operating room presented high risk of death. In the context of this study, staying in the operating room meant that patient couldn’t wake up early from GA and needed proper intensive care which couldn’t be acceded for due to lack of ICU infrastructure. Lack of admission or a late admission to ICU have been found as factors of high mortality postoperatively [38, 40, 63, 64]. Proper PACUs and ICUs should be implanted in theses hospital in order to decrease mortality.

Postoperative pain management was not implemented systematically and featured by the use of drugs with poor analgesia effect. This demonstrates a poor pain postoperative management and a need for improvement in this area of anaesthesia. Pain control is a core element of GA [3].

### Limitations

This study has provided data for the practice of GA in Butembo. As a baseline study, it will help further research in the field as a comparison where no previous data was available. The study covered most of the aspect of GA from preoperative period to postoperative period.

This study revealed that iv anaesthesia maintenance especially with Ketamine anaesthesia was safe in such low settings. The study has also revealed areas of improvement for safe practice of GA in the region. These are paediatric anaesthesia with emphasis on neonatal anaesthesia, postoperative anaesthesia care, intensive care management, enhance recovery after surgery, cardiopulmonary resuscitation and perioperative pain management. It has elicited a need for trained anaesthesia providers, appropriate drugs, equipment and infrastructure.

However, this study has some limitations. As a retrospective study some data were missing. This study used a simple logistic regression and no confounding factors were searched. As a baseline study in a low setting area, the objective was limited to determine all possible causes of death. Nevertheless, there is a need of further prospective research to elucidate and discriminate the factors. The definition of anaesthesia relation to death was only time linked and this study couldn’t state the cause of death. Although, it has been confirmed that anaesthesia complications occur early, there is a room to clarify this association to death by further research which will consider a peer review of each case of death to set the association to death.

## Conclusion

GA related mortality rate is high in Butembo but the trend is decreasing over time. Although the morbidity rate is high, it is comparable with other countries worldwide. Preoperative factors increasing mortality risk are the neonatal age and age equal or more than 50 years, emergency procedures, ASA PS (ASA equal or more than 2), single deranged vital signs at admission. Presenting a complication at any time intraoperatively was a factor increased risk of death. Intubation compared to non-intubation carried more risk of death. Cardiac arrest was a fatal complication. Postoperative deranged vital signs increased mortality. Presenting a complication postoperatively was a factor of death as well as being left in operating room as recovery place. Pain management was not adequate. There is a huge need to improve the practice of GA in the region by training more anaesthesia providers, proper patients monitoring, enhancing the infrastructure, equipment and drugs procurement and considering regional anaesthesia whenever possible.

## Data Availability

All data and material used in this article are available at any request from the corresponding author.

## Abbreviations

AMR: Avoidable mortality rate
ASA PS: American Association of Anesthesiologists Physical Status
COM: College of Medicine
COMREC: College of Medicine Research and Ethics Committee
CUG: Cliniques Universitaires du Graben
DRC: Democratic Republic of the Congo
EWS: Early warning score
GA: General Anaesthesia
ICU: Intensive Care Unit
PACU: Postoperative anaesthetic care unit
UCG: Université Catholique du Graben
