# Correction to: General anaesthesia related mortality in a limited resource settings region: a retrospective study in two teaching hospitals of Butembo

**DOI:** 10.1186/s12871-021-01297-7

**Published:** 2021-03-16

**Authors:** Furaha Nzanzu Blaise Pascal, Agnes Malisawa, Andreas Barratt-Due, Felix Namboya, Gregor Pollach

**Affiliations:** 1 Department of Anaesthesia and Intensive Care, College of Medicine, University of Malawi, Blantyre, Malawi; 2 Faculty of Medicine, Université Catholique du Graben, Butembo, Democratic Republic of the Congo; 3 Matanda Hospital of Butembo, Butembo, Democratic Republic of the Congo; 4 Division of Emergencies and Critical Care, Rikshospitalet, Oslo University Hospital, Oslo, Norway

**Correction to: BMC Anesthesiol 21, 60 (2021)**

**https://doi.org/10.1186/s12871-021-01280-2**

Following publication of the original article [1], the authors reported an error in Figs. 1 and 2 wherein the two figures were swapped and two typo errors. One typo is found under the Results section, 2nd sentence of the 3rd paragraph the word “*non-emergent”* was changed to *“non-emergency”* and the word “*that*” found in 1st sentence of the 5th paragraph under Discussion section was deleted.

The correct figures are as follows:

**Fig. 1:**
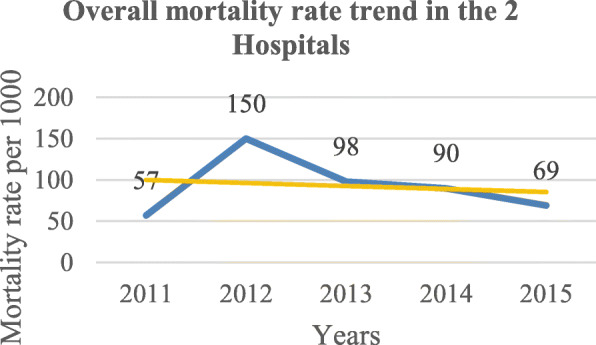
Overall mortality trend under GA in both hospitals

**Fig. 2:**
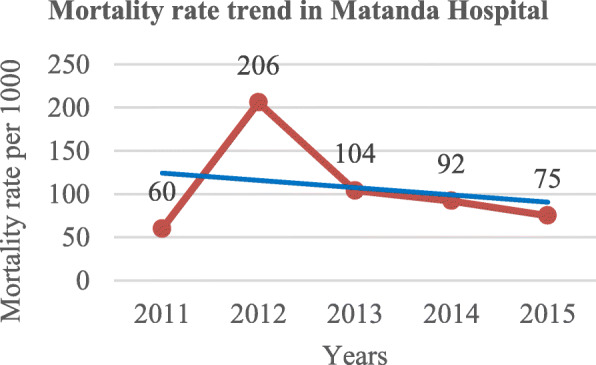
Overall mortality trend at Matanda Hospital

The original article has been corrected.
